# The Impact of Metolachlor Applications and Phytoremediation Processes on Soil Microorganisms: Insights from Functional Metagenomics Analysis

**DOI:** 10.3390/jox14030054

**Published:** 2024-07-23

**Authors:** Seyedeh Parvin Hejazirad, Caique Menezes de Abreu, Guilherme Henrique Fernandes Carneiro, Carlos Rodrigues Gomes, Paulo Roberto de Carvalho Spinola Filho, Márcia Regina da Costa, José Barbosa dos Santos

**Affiliations:** Department of Agronomy, Federal University of the Jequitinhonha and Mucuri Valleys, Diamantina 39100-000, Brazil; parvin.hejazirad@ufvjm.edu.br (S.P.H.); menezes.abreu@ufvjm.edu.br (C.M.d.A.); henrique.guilherme@ufvjm.edu.br (G.H.F.C.); carlos.gomes@ufvjm.edu.br (C.R.G.); spinola.filho@ufvjm.edu.br (P.R.d.C.S.F.); marcia.costa@ufvjm.edu.br (M.R.d.C.)

**Keywords:** herbicides, microbiome modulations, microbial biodiversity, phytoremediation, soil contamination

## Abstract

This study assessed the impact of phytoremediation on reducing the residual concentration of metolachlor in soil treated with doses of 530.7 and 1061.4 g/ha and its effect on microbial biodiversity in contaminated areas. For the plant species *Avena sativa* and *Medicago sativa*, a significant efficacy of 54.5 and 36.4% was observed in the dissipation of the herbicide, especially at higher doses. Although metolachlor application reduced soil microbial biodiversity, phytoremediating plants, especially *M. sativa*, promoted greater richness and distribution of microbial species, mitigating the negative effects of the herbicide. Principal component analysis revealed the influence of these plants and metolachlor on the composition of the microbial community. These results highlight the importance of phytoremediation in promoting soil biodiversity and reducing herbicide contamination, providing crucial insights for remediation strategies in contaminated areas.

## 1. Introduction

The extensive application of herbicides is a result of constant advancements in the studies of application technology and the expansion of cultivation areas, mainly for food, fiber, cellulose, and bioenergy production [1,2]. Several researchers have dedicated themselves to studying the impact of herbicides on the environment, obtaining differentiated responses regarding toxicity to non-target organism groups and the real effect on the sustainability of productive systems [3,4]. Reports of adverse effects on organisms and the functioning of soil ecosystems are observed [5], as well as persistence in soil and areas with accumulations of water, modulating interactions among non-target organisms, which is also considered negative [6].

The progress observed in recent decades regarding the development of safer molecules and the reduction of product losses to the environment through innovation in herbicide application techniques is undeniable [7].

Approaches to the impact of herbicide use have strongly considered the negative impact of molecules on soil microorganisms [7]. However, a very small portion of research discusses with authority the concentrations used, as well as the final effect on soil microbial dynamics [8,9].

Considering tropical agriculture during grain cultivation, we observe the use of two groups of herbicides, one applied pre-emergence and the other post-emergence of plants. These herbicide groups complement each other in weed control, as while one acts on the seed bank and prolongs the effect over time, the other acts directly on treated plants, controlling present vegetation and ensuring the formation of organic material through plant residue [10,11]. The mixture of metolachlor and glyphosate herbicides has these working effects, and for this reason, it was chosen for this research.

Metolachlor(2-chloro-N-(2-ethyl-6-methylphenyl)-N-[(1S)-2-methoxy-1-methyl] acetamide) is a herbicide belonging to the chloroacetamide class. In the United States, metolachlor is among the three herbicides with the highest volume of use [12]. It has prolonged persistence in the soil, commonly used in crops such as *Eucalyptus* sp., *Zea mays* L., *Glycine max* (L), *Solanum tuberosum* L., *Beta vulgaris* L., *Helianthus annuus* L., *Solanum lycopersicum* L., and various others, as well as on lawns.

In several agriculturally important countries, metolachlor has been particularly effective against *Amaranthus* sp. *Amaranthus palmeri* was well controlled in corn crops in the United States with metolachlor [13]. In Brazil, metolachlor has been one of the main herbicides applied pre-emergence, mainly for grass and some broadleaf weed control [14,15]. In Brazil, *Amaranthus palmeri* is the main pest listed by the Ministry of Agriculture [16], and metolachlor is one of the control options. The applied doses of metolachlor average 960 g/ha for weed control in grain legume crops [17]. A study indicates that the safe application of metolachlor at a rate of 480 g/ha can be applied approximately 20 days before crop sowing to control *Lolium rigidum* in wheat selectively [18]. Thus, the dosage of metolachlor may vary depending on factors such as the specific crop, targeted weeds, soil clay content, organic matter, and local conditions and regulations [19]. The herbicide’s persistence, which refers to its control power for a longer period, is highly dependent on climate and soil conditions [20]. Under laboratory conditions, metolachlor has higher persistence [21,22]. Thus, research should consider field-observed results as being more promising.

An innovative approach advocated in this research is the study of the impact of metolachlor on soil microbial dynamics. Research on the impact of herbicides on microorganisms is constantly conducted, but innovations in integrated weed management have shifted the perspective of the hypotheses tested. Phytoremediation green technology is a good example of research where the potential for microbial degradation is associated with remedial species tolerant to herbicide residues [23]. The adoption of phytoremediators in modern agriculture has advanced significantly, with a strong trend towards increasing use due to the need for better utilization of tropical agricultural areas, with incentives for efficient crop rotation [15,24]. In this sense, it is necessary to know species adapted to summer or winter to protect fields between main crops, covering the soil and competing with weeds while reducing residues from herbicides previously applied pre-emergence [25,26]. Therefore, phytoremediation is an innovation desired by farmers who wish to maintain the positive effect of residual herbicides while eliminating the impact on sensitive crops, known as the carryover effect [24,27].

A highly debated issue regarding the future use of herbicides is the impact of these molecules on agricultural sustainability. There is a contradiction between implementing phytoremediation technologies to allow for the rational use of pre-emergence herbicides and the expectation of greater preservation of microbial diversity, as herbicides are considered harmful to microorganisms. To assist in studies of this complexity, genetic studies have been proposed to comprehensively evaluate the real impact of herbicides on soil health, especially in intensive agriculture systems such as tropical ones [24,28].

Studies on metagenomics have been used to identify taxonomic and functional groups of microorganisms, genes, and metabolic pathways associated with xenobiotic manipulation, which typically involve an assessment of the microbiota and the identification of markers indicating the presence of individuals, genes, and enzymes involved in the manipulation process [23,29]. Herbicide application in soil can result in various effects on different components of the soil microbial community, with both beneficial and adverse consequences. The nature of these effects, whether direct or indirect, determines the overall impact on the microbial community [30]. The consequences involve herbicides being detrimental to microorganisms, leading to a decrease in microbial biomass, subsequently affecting soil heterotrophic respiration, organic matter decomposition, and the activity of microbes involved in the nutrient cycle [31,32]. On the other hand, herbicide addition can have a direct positive effect on soil microbes, providing a resource that supports their growth, for example, by inhibiting some less important groups in soil resilience [33]. Additionally, the impact of herbicides on plants can indirectly influence microorganisms [34].

We hypothesize that the phytoremediation process, considered a green technology for tropical agriculture, may be key in the relationship between herbicide residues and soil health as measured by functional genomics of the plant rhizosphere-associated microbiota. Thus, the objective of this study was to analyze the impact of using metolachlor as a case study of herbicides and phytoremediating plants on soil biodiversity and resilience.

## 2. Materials and Methods

The experiment was conducted in the field at the experimental area of the Faculty of Agricultural Sciences of the Federal University of the Valleys of Jequitinhonha and Mucuri—UFVJM, located at the JK campus, Diamantina, MG. The geographic coordinates of the area are latitude 18°10′ S and longitude 43°30′ W, with an altitude of 1388 m. The region has a mesothermal climate, classified as a *Subtropical high-altitude—Cwb* according to the Köppen classification, characterized by mild and humid summers and cold and dry winters. Before the experiment, the area was desiccated with glyphosate (1440 g/ha) and cultivated with corn and beans without the application of pre-emergence herbicides.

The soil where the experiment was installed is classified as Orthic Quartzarenic Neosol, with a sandy loam texture. Its chemical characteristics include a pH (H_2_O) of 5.20, 2.64 cmolc dm^−3^ of H + Al^+^, 1.19 cmolc dm^−3^ of Ca^2+^, 0.15 cmolc dm^−3^ of Mg^2+^, 8.10 mg dm^−3^ of P, 36.0 mg dm^−3^ of K, and 1.60 dag kg^−1^ of organic matter [35]. The experiment was conducted in a randomized complete block design with three replications.

Ten treatments were planned to combine doses of metolachlor (in the ready mix with glyphosate, called Sequence^®^) and two green manure species, selected according to their potential for remediating areas with herbicides: *A. sativa* (cultivate: URS-Taura/BRSEE-Ds^®^) [36] and *M. sativa* (cultivate: URS/BRSEE-Ds^®^) [37]. The list of treatments is presented in Table 1.

After soil preparation and fertilization, commercial recommendations for green manures were followed [38,39]. The commercial mixture Sequence^®^ was applied, composed of metolachlor (353.8 g/L) and glyphosate (265.7 g/L). For this research, we did not consider the effect of glyphosate due to its strong soil adsorption [40]. So, the treatments were conditioned to the residual effect of metolachlor. We opted for metolachlor doses, equivalent to half (1.5 L/ha) and the full average (3.0 L/ha) recommended dose for major agricultural and forestry crops. For application, a backpack sprayer equipped with a transversal bar with four nozzles at a 110° angle and a flow rate of 0.15 gallons per minute, equivalent to 150.0 L ha^−1^ of solution, was used. Three days after application, the predetermined plots were sown with the species *A. sativa* and *M. sativa.* The planting density was 7.0 g and 2.0 g seeds/m^2^, respectively, for *A. sativa* and *M. sativa* [36,37].

**Microbial community metagenome**

After 60 days of herbicide application, three plants were sampled per plot, and the roots were removed to collect the rhizosphere soil. These samples were collected and kept at −80 °C for metagenomic analysis. Samples were taken only from plots growing *A. sativa* and *M. sativa*, with and without herbicide, and from native vegetation soil with no previous herbicide contamination (No-0). Metagenomic DNA was extracted and purified. Sequencing was performed on the Illumina Hiseq 2000 platform (Illumina, San Diego, CA, USA) with amplification of the 16S rRNA and ITS genes. Data pre-filtering was performed using the Readfq program v8 https://github.com/cjfields/readfq (accessed on 22 January 2024) to remove sequences containing low-quality bases and those that did not reach a proportion greater than 10.0%. Subsequently, high-quality sequences from each sample were assembled (MEGAHIT), generating “Scaffolds” with fragments larger than or equal to 500 bp. The sequences were compared to the genomic database of bacteria, fungi, and archaea using the Blastx tool. Genetic sequences were processed and compared with the database of fungi and bacteria to identify and quantify the species present in the samples. The relative abundance values (%) of microorganisms were determined by the proportion of the microorganism among all the groups to which it belongs. The symbol (*) represents the microbial group below the top 10 occurrences of microorganisms at the family and genus levels.

The domain was estimated using the Gini-Simpson indices [41], and the richness was quantified by the number of different species present in the sample, according to Chapo1. The species distribution quantified the diversity based on the number of species present in the samples, and for this, we used the Shannon Index [42]. Additionally, to indicate the distribution of the different species in the sample, we estimated using the Pielou index, determining the uniformity [43]. The diversity of soil microorganisms was estimated as a percentage based on dominance, richness, species distribution, and uniformity between species indices. To determine correlations between rhizosphere microbiota, phytoremediating plants, and herbicide doses, principal component analysis, Pearson correlation network in cords, and neural networks were performed using R Software v4.4.1.

Principal component analysis (ACP) of microbial biodiversity was performed based on the microbial phyla associated with phytoremediating plants to determine changes in the studied samples. The separation of groups and subgroups was determined by the significance cutoff point (above 0.9 or below −0.9). The distribution of microbial phyla for the plants *A. sativa* and *M. sativa* was determined with the Pearson correlation network (above 0.6 or below −0.6) and grouped by the string procedure established according to the groups formed by the ACP. The distribution of microbial families associated with *A. sativa* and *M. sativa* plants in soil phytoremediation areas containing metolachlor residues was estimated with Pearson’s correlation with a significance cutoff point (above 0.6 or below −0.6), represented graphically by the neural network.

**UHPLC MS/MS analysis residue of the herbicide**

After 80 days of herbicide application, soil samples were collected to determine herbicide residue in the soil by chromatography. Samples were collected from plots with herbicide application, i.e., plots with remediation species and uncultivated areas that received herbicide application and were weeded throughout the period. For the analytical determination of residues, extraction was performed by the modified QuEChERS method [44], and analysis was carried out by ultra-high-performance liquid chromatography coupled with tandem mass spectrometry (UHPLC-MS/MS). The extracts were cleaned using dispersive solid-phase extraction (d-SPE). For the d-SPE phase, magnesium sulfate and the sorbents octadecylsilane (C18) and primary secondary amine (PSA) were used. The extracts were then shaken, centrifuged, and filtered. Before analysis by UHPLC-MS/MS, the samples were diluted five times in ultrapure water. The samples were analyzed on the Waters (USA) UHPLC-MS/MS system, equipped with a liquid chromatograph; triple quadrupole MS detector, model Xevo TQ; electrofoil ionization source/interface; nitrogen peak generator; solvent control system (binary pump system) for high-pressure gradient operation; analytical column Acquity UPLC^®^ BEH C18 (50 × 2.1 mm, 1.7 μm) from Waters (USA); and data acquisition system with MassLynx software v4.1 (Waters, Milford, MA, USA). Selected reaction monitoring was used for quantification and identification of substances to be analyzed. The mobile phase used was (A) water:methanol (98:2, *v*/*v*) and (B) methanol, both containing 5 mmol L^−1^ of ammonium format and 0.1% formic acid (*v*/*v*), with a flow rate of 0.225 mL min^−1^ and an injection volume of 10 µL [45]. The manipulation estimate was determined by the average value of each area for a qualitative analysis. The values presented in the figures are given as means ± standard errors.

## 3. Results

The remediation species *Avena sativa* and *Medicago sativa* exhibited satisfactory growth in the areas, regardless of metolachlor residues, confirming their tolerance to this herbicide.

**Soil Bioremediation**

**Figure 1:**
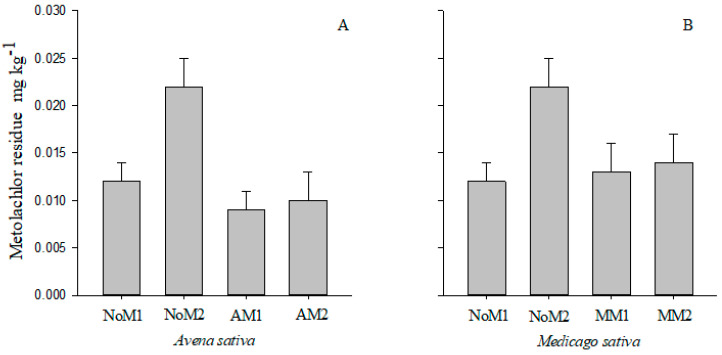
Metolachlor residue, 80 days after application of two doses (530.70 and 1061.40 g/ha) in areas cultivated or not cultivated with *Avena sativa* (**A**) and *Medicago sativa* (**B**). (NoM1-No plants + 530.70 g/ha; NoM2-No + 1061.40 g/ha; AM1-*A. sativa* + 530.70 g/ha; AM2-*A. sativa* + 1061.40 g/ha; MM1-*M. sativa* + 530.70 g/ha; MM2-*M. sativa* + 1061.40 g/ha). The values presented in the figures are given as means ± standard errors.

Soil remediation containing metolachlor was observed in areas managed with green manures *A. sativa* (AM1 and AM2) and *M. sativa* (MM1 and MM2) for 80 days, where the residual concentration in the soil depends on the remediation species (Figure 1A,B). Remediation efficiency was particularly increased in areas AM2 and MM2, where the herbicide application dose was 1061.40 g L of metolachlor (Figure 1A,B). Residual concentrations of metolachlor were 45.5% (NoM1) and 75.0% (NoM2), at 0.012 mg kg^−1^ and 0.022 mg kg^−1^, respectively, dissipating 25.0% (AM1) and 54.5% (AM2) of the molecule in the presence of *A. sativa* (Figure 1A). In the presence of *M. sativa*, there was a dissipation of 7.7% (MM1) and 36.4% (MM2) of the soil molecule (Figure 1B). The dynamics of soil microbiota in association with phytoremediating plants were diverse and selective, with the potential for herbicide molecule biodegradation at high concentrations in areas AM2 and MM2.

**Global Biodiversity and Microbiome Composition**

In the control soil sample (from Cerrado) where there was a concentration of 1061.40 g/ha of metolachlor and persistence of 0.012 (AM1), 0.014 (AM2), 0.009 (MM1), and 0.0010 (MM2) mg/kg^−1^ of the molecule, there was high microbial biodiversity (Table 2).

Soil samples A0 and AM1 showed lower species distribution uniformity according to the Pielou index, reaching −17.44% compared to areas with medium and high herbicide concentrations (Table 2). For *A. sativa*, the richness of the associated microbial species was higher in AM1 and AM2, with 116–121 species. The distribution of microbial species in area A0 was 19.51% lower than in AM2 and No-0. In *M. sativa*, the highest richness was observed in M0 and MM1, ranging from 5 to 13 compared to MM2 and No-0. *M. sativa* exhibited higher values of evenness, richness, distribution, and dominance compared to *A. sativa* (Table 2). In the MM1 areas, there was a 10.6% greater richness compared to the No-0 area. In the MM2 treatment, richness decreased by 4.1% compared to the MM1 area. The distribution of species in the native Cerrado area was 4.8% lower than in the rhizospheric environment of *M. sativa*, regardless of metolachlor presence (Table 2). For *M. sativa*, the response to dominance was the same, regardless of the treatment. Overall, the values for the global ecological index indicate the following decreasing sequence for biodiversity: MM1 (80.0%) > M0 (79.1%) > No-0 (62.83%) > A0 (61.9%) > MM2 (61.06%) > AM2 (60.17%) > AM1 (59.29%).

**Figure 2:**
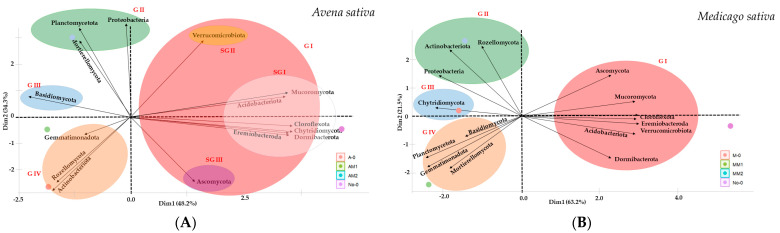
Principal component analysis (PCA) of microbial biodiversity associated with *Avena sativa* (**A**) and *Medicago sativa* (**B**) in metolachlor residual areas. A0-*A. sativa* in soil without herbicide (control), AM1 and AM2-*A. sativa* cultivated in soil with 530.70 and 1061.40 g/ha of metolachlor. No-0-native Cerrado soil. M0—*M. sativa* in soil without herbicide (control), MM1 and MM2-*M. sativa* cultivated in soil with 530.70, and 1061.40 g/ha of metolachlor. GI-grouping I; GII-grouping II; GIII-grouping III; GIV-grouping IV; SGI-subgroup I; SG II-subgroup II; SG III-subgroup III.

**Figure 3:**
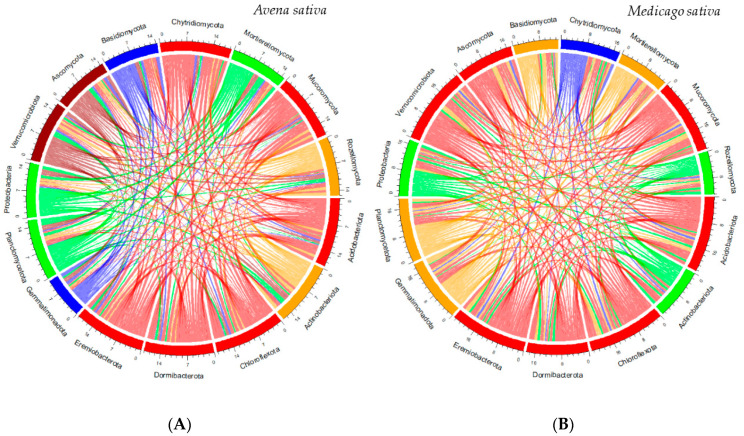
Distribution of microbial phyla associated with *Avena sativa* (**A**) and *Medicago sativa* (**B**) in soil phytoremediation areas containing metolachlor residues. The correlation networks in cords were grouped using the Pearson procedure, and the coloring was established according to the groups formed by the PCA. Red: Group I; Green: Group II; Blue: Group III; Orange: Group IV.

The principal component analysis of soil phytoremediation with *A. sativa* and *M. sativa* highlighted the relationships between the abundance value of microbial phyla concerning herbicide doses and uncultivated Cerrado soil, forming four phylogenetic groups (Figure 2A,B). Regarding *A. sativa*, native Cerrado soil samples (No-0) formed cluster I, with three subgroups. Subgroup I is composed of Acidobacteriota, Chloroflexota, Chytridiomycota, Dormibacterota, Eremiobacterota, and Mucoromycota (Figure 2A). Subgroup II is formed by Verrucomicrobiota, and subgroup III by Ascomycota (Figure 2A). Cluster II, related to the environment with higher metolachlor dose, is formed by Planctomycetota, Proteobacteria, and Mortierellomycota. Cluster III, related to the lower metolachlor dose, is formed by Basidiomycota, and cluster IV, *A. sativa* without herbicide, is formed by Rozellomycota, Gemmatimonadota, and Actinobacteria. For *M. sativa*, cluster II, also related to the higher herbicide dose, was formed by Actinobacteria, Proteobacteria, and Rozellomycota. Cluster III, *M. sativa* without herbicide, was formed solely by Chytridiomycota. Cluster IV, regarding the lower herbicide dose, was formed by Basidiomycota, Gemmatimonadota, Planctomycetota, and Mortierellomycota (Figure 2B). The percentage of the interaction of microbial phyla in the rhizospheric soil of *A. sativa* was 28.8%, highly positive (>0.6) among the 15.0 observed phyla (Figure 3A). Null interactions (0.0), moderately correlated (between 0.3 and 0.5), and highly negative correlations (<0.6) were, respectively, 0.5%, 56.6%, and 14.2%, highlighting the dynamism among organisms (Figure 3A). Positive correlations between microbial phyla with *M. sativa* were 1.5 times lower compared to *A. sativa* (Figure 3B). In rhizospheric soils of *M. sativa*, microorganisms of the phyla Mortierellomycota x Dormibacterota, Gemmatimonadota x Actinobacteria, and Ascomycota x Rozellomycota show null correlations (Figure 3B).

**Figure 4:**
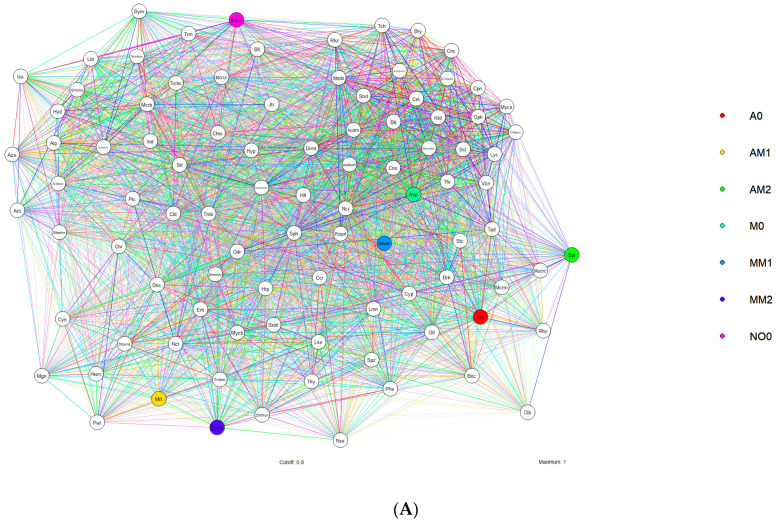

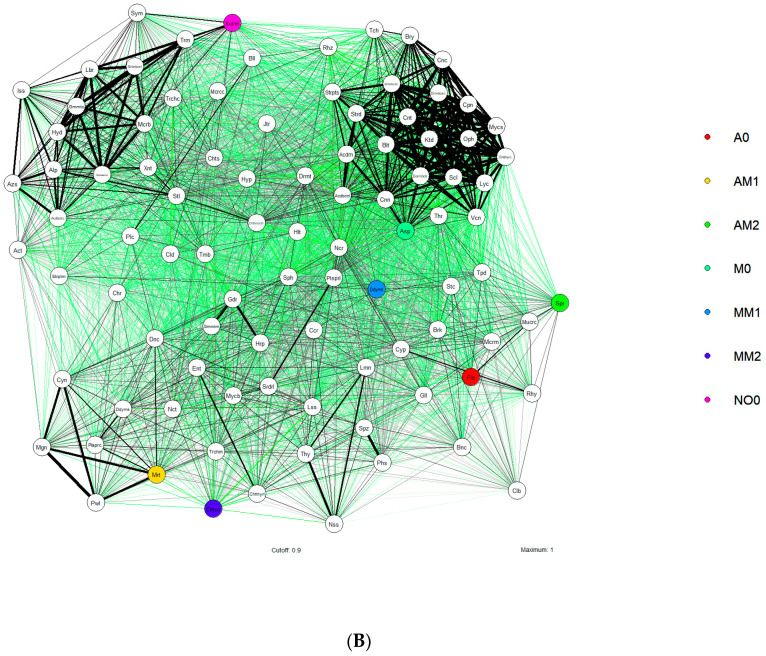
Distribution of microbial families associated with *Avena sativa* (**A**) and *Medicago sativa* (**B**) in soil phytoremediation areas containing metolachlor residues. The colors mark the point of highest correlation between treatments and organisms: red: A0, yellow: AM1; green: AM2; light green: M0; blue: MM1; purple: MM2; and pink: No-0. The neural correlation networks were clustered using the Pearson procedure with a significance cutoff (>0.6). *Ace-Acetobacteraceae*, *Aci-Acidimicrobiales*, *Acd-Acidobacteriaceae*, *Acdb-Acidobacteriales*, *Alp-Alphaproteobacteria*, *Asp-Aspergillaceae*, *Azs-Azospirillaceae*, *Blt-Baltobacteraceae*, *Bnc-Bionectriaceae*, *Brt-Bryobacteraceae*, *Bll- Bulleribasidiaceae*, *Brk-Burkholderiaceae*, *Cpn-Capnodiales*, *Clb-Caulobacteraceae*, *Cht-Chaetomiaceae*, *Chts-Chaetosphaeriaceae*, *Chtt-Chaetothyriaceae*, *Chtth-Chaetothyriales*, *Chr-Chrysozymaceae*, *Chtho-Chthoniobacteraceae*, *Chthn-Chthoniobacterales*, *Cld-Cladosporiaceae*, *Cnc-Coniochaetaceae*, *Cnt-Coniothyriaceae*, *Ccr-Cucurbitariaceae*, *Cnn-Cunninghamellaceae*, *Cyn-Cyanobacteriia*, *Cyp-Cyphellophoraceae*, *Dnc-Deinococcaceae*, *Drm-Dermatophilaceae*, *Ddy-Didymellaceae*, *Ddym-Didymosphaeriaceae*, *Drm-Dormibacteraceae*, *Drmb-Dormibacteria*, *Ent-Enterobacteriaceae*, *Flb-Filobasidiaceae*, *Gll-Gaiellaceae*, *Gmm-Gemmataceae*, *Gmmt-Gemmatimonadaceae*, *Gdr-Geodermatophilaceae*, *Helt-Helotiaceae*, *Hrp-Herpotrichiellaceae*, *Hydn-Hydnodontaceae*, *Hypc-Hypocreaceae*, *Iss-Isosphaeraceae*, *Jatr-Jatrophihabitantaceae*, *Ktd-Ktedonobacteraceae*, *Lbr-Labraceae*, *Lss-Lasiosphaeriaceae*, *Lmn-Limnocylindrales*, *Lyc-Lycoperdaceae*, *Mgn-Magnaporthaceae*, *Mcr-Microbacteriaceae*, *Micr-Micrococcaceae*, *Micrm-Micromonosporaceae*, *Mort-Mortierellaceae*, *Mucr-Mucoraceae*, *Myc-Mycobacteriaceae*, *Mycs-Mycosphaerellaceae*, *Nect-Nectriaceae*, *Nss-Niessliaceae*, *Ncr-Nocardioidaceae*, *Oph-Ophiocordycipitaceae*, *Phsp-Phaeosphaeriaceae*, *Pltc-Plectosphaerellaceae*, *Pls-Pleosporaceae*, *Plsp-Pleosporales*, *Pow-Powellomycetaceae*, *Rhi-Rhizobiaceae*, *Rhy-Rhynchogastremataceae*, *Scl-Sclerotiniaceae*, *Slr-Solirubrobacteraceae*, *Sor-Sordariomycetes*, *Sph-Sphingomonadaceae*, *Spz-Spizellomycetales*, *Spr-Sporidiobolaceae*, *Stc-Stachybotryaceae*, *Stl-Stellaceae*, *Str-Steroidobacteraceae*, *Strep-Streptomycetaceae*, *Streptos-Streptosporangiaceae*, *Sym-Sympoventuriaceae*, *Teic-Teichosporaceae*, *Tep-Tepidisphaeraceae*, *Thr-Thermoleophilia*, *Thy-Thyridariaceae*, *Trchc-Trichocomaceae*, *Trchom-Trichomeriaceae*, *Tricm-Trimorphomycetaceae*, *Tum-Tumebacillaceae*, *Vic-Vicinamibacterales*, *Xan-Xanthobacteraceae*.

A total of 94 families of fungi and soil bacteria were found to be associated with *A. sativa* and *M. sativa* in the presence and absence of the herbicide molecule: *Acetobacteraceae*, *Acidimicrobiales*, *Acidobacteriaceae*, *Acidobacteriales*, *Alphaproteobacteria*, *Aspergillaceae*, *Azospirillaceae*, *Baltobacteraceae*, *Bionectriaceae*, *Bryobacteraceae*, *Bulleribasidiaceae*, *Burkholderiaceae*, *Capnodiales*, *Caulobacteraceae*, *Chaetomiaceae*, *Chaetosphaeriaceae*, *Chaetothyriaceae*, *Chaetothyriales*, *Chrysozymaceae*, *Chthoniobacteraceae*, *Chthoniobacterales*, *Cladosporiaceae*, *Coniochaetaceae*, *Coniothyriaceae*, *Cucurbitariaceae*, *Cunninghamellaceae*, *Cyanobacteriia*, *Cyphellophoraceae*, *Deinococcaceae*, *Dermatophilaceae*, *Didymellaceae*, *Didymosphaeriaceae*, *Dormibacteraceae*, *Dormibacteria*, *Enterobacteriaceae*, *Filobasidiaceae*, *Gaiellaceae*, *Gemmataceae*, *Gemmatimonadaceae*, *Geodermatophilaceae*, *Helotiaceae*, *Herpotrichiellaceae*, *Hydnodontaceae*, *Hypocreaceae*, *Isosphaeraceae*, *Jatrophihabitantaceae*, *Ktedonobacteraceae*, *Labraceae*, *Lasiosphaeriaceae*, *Limnocylindrales*, *Lycoperdaceae*, *Magnaporthaceae*, *Microbacteriaceae*, *Micrococcaceae*, *Micromonosporaceae*, *Mortierellaceae*, *Mucoraceae*, *Mycobacteriaceae*, *Mycosphaerellaceae*, *Nectriaceae*, *Niessliaceae*, *Nocardioidaceae*, *Ophiocordycipitaceae*, *Phaeosphaeriaceae*, *Plectosphaerellaceae*, *Pleosporaceae*, *Pleosporales*, *Powellomycetaceae*, *Rhizobiaceae*, *Rhynchogastremataceae*, *Sclerotiniaceae*, *Solirubrobacteraceae*, *Solirubrobacterales*, *Sordariales*, *Sordariomycetes*, *Sphingomonadaceae*, *Spizellomycetales*, *Sporidiobolaceae*, *Stachybotryaceae*, *Stellaceae*, *Steroidobacteraceae*, *Streptomycetaceae*, *Streptosporangiaceae*, *Sympoventuriaceae*, *Teichosporaceae*, *Tepidisphaeraceae*, *Thermoleophilia*, *Thyridariaceae*, *Trichocomaceae*, *Trichomeriaceae*, *Trimorphomycetaceae*, *Tumebacillaceae*, *Vicinamibacterales*, and *Xanthobacteraceae* (Figure 4A,B).

The families of microorganisms responded satisfactorily to the biotic and abiotic pressure imposed by soil agricultural disturbance, even with the application of metolachlor and the cultivation of green manures (Figure 4).

**Ecological indices of bacterial population in soil**

The uniformity of bacterial species distribution in the rhizosphere of *A. sativa* was different from the other treatments for this species (Table 3). In areas cultivated with *A. sativa* containing metolachlor residues, all ecological indices showed reductions—29.0%, 32.0%, 33.0%, and 24.0%, respectively, for uniformity, richness, distribution, and dominance (Table 3). This effect was not observed in soil samples from the area with *M. sativa* cultivation (Table 3).

The abundance of bacteria varied among treatments, with the most prevalent families presented in Table 4. Families with abundance greater than 10.0% were *Burkholderiaceae* (10.63% for A0), *Micrococcaceae* (11.01% for A0, 10.2% for AM1, 14.27% for AM2, and 36.6% for M0, 17.5% for MM1), and *Xanthobacteraceae* (12.56% for AM2, and 17.74% for MM2). The observed *Bryobacteraceae* family was less abundant in areas with *M. sativa*. Similarly, *Ktedonobacteraceae* was not observed in areas with *A. sativa* (Table 4).

Despite the low percentage of undetermined families (typically new ones or those with DNA fragments insufficient for cataloging), the highest percentage of abundance for predominant bacterial genera was considered undetermined, ranging from 31.0% (for M0) to 47.12% (for A0). Also, a higher percentage of uncultivated bacterial genera were observed, composed of species with ecological importance but difficult to cultivate in vitro (Table 4). The most predominant bacterial genera were *Bradyrhizobium* sp. (4.78%), *Massilia* sp. (2.06%) in AM2; *Sphingomicrobium* sp. (2.69%), *Mycobacterium* sp. (2.29%), *Arthrobacter* sp. (1.2%), *Pseudarthrobacter* sp. (0.8%) in AM1; and *Pseudarthrobacter* sp. (3.47%), *Streptomyces* sp. (2.59%), *Sphingomicrobium* sp. (2.01%), *Baekduia* sp. (0.89%) in A0.

**Ecological indices of the fungal population in the soil**

The uniformity of fungal species distribution also varied to a greater degree in the rhizosphere of *A. sativa* compared to *M. sativa* (Table 5). The values were lower for soil samples of *A. sativa* without metolachlor and under the effect of the lower herbicide dose. A similar effect was observed for the distribution variable (Table 5). Regarding *M. sativa*, the values for uniformity, richness, distribution, and dominance were relatively consistent among treatments; however, they were higher than those observed for native Cerrado soil (Table 5).

Among the abundance percentages of fungal families, the highest average values (above 10.0%) were observed for *Aspergillaceae* (10.02%), *Didymellaceae* (10.05%) in *A. sativa* soils, and *Chaetomiaceae* (10.27%) and *Nectriaceae* (17.71%) in *M. sativa* soils (Table 6). Among these averages, the highest percentages observed were for *Aspergillaceae* in AM2 (12.18%), *Didymellaceae* in AM2 (13.19%), *Chaetomiaceae* in MM2 (13.69%), and *Nectriaceae* in M0 (28.88%) (Table 6). In the assessment of each treatment, other high values were observed for *Mortierellaceae* in AM1 (19.57%), *Aspergillaceae* in M0 (10.49%), *Cladosporiaceae* in M0 (10.06%%) and MM1 (14.61%), *Didymellaceae* in M0 (13.84%), and, with the greatest discrepancy, *Rhynchogastremataceae* in MM1 (20.0%) (Table 6). Under native Cerrado soil, the highest percentage of abundance was observed for *Aspergillaceae* (21.14%) and *Nectriaceae* (18.60%). The percentages of the abundance of undetermined fungal families were higher for AM1 (17.35%), AM2 (18.13%), and Cerrado native soil (11.06%) (Table 6).

No significant percentage of the families *Filobasidiaceae* and *Rhynchogastremataceae* was observed in soil samples of *A. sativa*. On the other hand, *Mortierellaceae* did not show significant abundance in soils with *M. sativa* (Table 6).

The genera *Fusarium* sp. and *Penicillium* sp. were observed with higher abundance in the rhizosphere of *A. sativa*; however, their presence was significantly reduced in the presence of metolachlor (Table 6). The genera *Cladosporium* sp. and *Papiliotrema* sp., also abundant in *A. sativa*, showed higher activity and abundance under the effect of the lower herbicide dose; however, their occurrence decreased with the increase in dose. In *M. sativa*, the occurrence of *Papiliotrema* sp. and *Saitozyma* sp. was not significant. On the other hand, *Fusarium* had an abundance percentage of 11.94 in the rhizospheric soil of *M. sativa* but decreased in the presence of the herbicide (Table 6).

## 4. Discussion

Despite being tolerant to herbicides, the tested species showed divergent potential for remediation. The more effective soil exploration by grasses results in a higher rate of nutrient and molecule absorption [43], which could explain the higher efficiency of *A. sativa*. This species has an optimized and fasciculate root length in the soil, allowing for the exploration of a larger volume of particles with molecule sorption. Its tolerance to metolachlor and various other herbicides has already been reported [46].

Due to its taproot system, *M. sativa* has secondary roots that can intercept the molecule to be remediated in lower concentrations. However, the presence of exudates acting in the remediation process should be considered. Compounds such as alginate oligosaccharides are beneficial for alfalfa root growth [47]. According to Ref. [48], many herbicides have been formulated with different carriers, including alginate, which allows for greater sorption.

In both situations, plants can select communities of microorganisms to compose the rhizospheric system, and their interaction can have a direct impact on the extent of herbicide degradation [49]. The interaction between microorganisms and plants remediating environments with herbicide residues is underexplored, but it can be synergistic and positive [50].

The herbicide should be strictly applied according to the regimen that defines its optimal dosage to mitigate any potential disturbance in soil homeostasis. A dose of 1.200 g of metolachlor can contribute to the increase in soil dehydrogenase activity promoted by the microbiota [51]. In overdoses ranging from 15 to 400 g per kg of soil, the dynamics and ecological state of the soil are modified [52].

The high uniformity of species distribution in environments AM2, No-0, and AM1 demonstrates that the herbicide is not capable of interfering with nutritional consumption. Under controlled conditions, it was found that part of the microbial energy consumption can come from the content of the metolachlor molecule, which has 15 carbon atoms and one nitrogen atom [53]. On the other hand, microbial specificity or restriction is more affected by the rhizospheric effect of *A. sativa* than by the presence of the herbicide. The restrictive association, due to microbial specificity, has already been observed for *A. sativa* [54], and the herbicide seems to provide greater abundance to the rhizosphere.

The presence *of A. sativa* modulates the richness of the microbial population. This result leads to the hypothesis that the presence of the herbicide in the soil stimulated the formation of a more diverse community (Table 2). Practically speaking, our findings indicate soils that are more diverse and balanced, with a dominance between 0.92 and 0.97 according to the Gini-Simpson index. In other words, an equitable and resilient rhizospheric microbial community signals soil with ecological health in all treatments (Table 2).

Bacterial and fungal richness increases during the early stages of *M. sativa* cultivation [55]. The presence of the herbicide appears to interfere with rhizosphere dynamics, promoting a greater number of species. Although this increase is less apparent at higher concentrations, it still surpasses the richness observed in the absence of the molecule.

The reduction in species distribution in native Cerrado soil indicates that opting for legume cultivation will promote a highly resilient, stable, and balanced microbial population, evidencing the conditioning of healthy soil with a wide variety of ecological niches, even with the likely interaction with herbicide residues, after 80 days of application (Table 2).

Thus, regarding the integration of the ecological indices analyzed, we can infer that the presence of the legume (*M. sativa*) provides greater microbial biodiversity, surpassing the environment cultivated with *A. sativa* and the native Cerrado environment. However, under the effect of the dose of 1061.4 g/ha of metolachlor, this biodiversity equals the other treatments, indicating that recommendations for *M. sativa* cultivation may be interesting in areas where metolachlor is used if the practiced doses are low.

In our research, the principal component analysis highlights groups with greater similarity to the imposed treatment [56] in the cultivation of *A. sativa* and *M. sativa* with the presence of metolachlor. Overall, the cultivation of these species groups of microbial phyla isolates a special grouping for native Cerrado soil. It has been observed that native tropical soils exhibit greater microbial diversity compared to systems that promote disturbance, such as those of intense agriculture [57]. Our findings have already proven that cultivation with green manures such as *A. sativa* and, especially, *M. sativa*, can mitigate negative effects, maintaining good biodiversity of microbial species, even with low doses of metolachlor. For phylogenetic diversity, the formation of groups that demonstrate the effect of agricultural activity is clear, separating, to a greater extent, the native Cerrado environment. The variation in the abundance of rare species “depends on rare microbes” and is linked to changes in anthropized areas. For this reason, they are observed in environments with low agricultural activity [58]. However, they are important because they drive key processes in geochemical cycles and are significant actors in the degradation of xenobiotics.

The behavior of common microorganisms remains consistent regardless of the cultivated species, with groups observed for the species without herbicide or with the lowest dose and a new group for samples under higher doses of metolachlor. Thus, the differentiated effect for areas with the highest dose of the herbicide is confirmed.

We verified through principal component analysis that phyla such as Proteobacteria are present in the environment with higher doses of metolachlor for both plant species. However, the phyla Planctomycetota, Mortierellomycota, and Basidiomycota are examples of groups that tolerate the herbicide at higher doses in the rhizospheric environment of *A. sativa* but not for *M. sativa*, indicating a remedial action resulting from the possible plant–microorganism interaction. Similarly, the phyla Rozellomycota and Actinobacteriota were significant in the rhizosphere of *M. sativa* under the effect of the highest herbicide dose; however, in *A. sativa*, this was only under the effect of the highest dose or in the absence of the herbicide. Our findings are relevant for selecting groups of microorganisms to produce bioinputs used in the decontamination of areas, also because the recommendation of the microbial group is highly dependent on the remediating plant species.

The distribution of phyla occurs depending on the phytoremediating plant. The herbicide possibly moderates it, as the phylum Rozellomycota was highly correlated with Ascomycota in the presence of *M. sativa*, indicating synergy between the organisms (Figure 3A). This synergy may explain the remediation potential of *M. sativa*. Soils with abundant fungi have been reported as promising for the rapid degradation of metolachlor [59].

Despite agricultural activities causing disturbances in the soil environment, our findings demonstrate that the cultivation of green manures improves soil organic matter and increases microbial richness, especially when considering distinct families such as grasses and legumes [60]. These microbial communities, associated with phytoremediators, play a fundamental role in the degradation of organic compounds and contribute to the resilience of the ecosystem, even in agricultural systems with pre-emergent herbicide applications such as metolachlor.

The pressure exerted by agricultural cultivation and herbicide application has altered the interaction between organisms, where rare groups in lower abundance are essential in soil suppressiveness and homeostasis [57]. The negative impact of metolachlor on microorganisms has been reported for in vitro experiments and at doses above those practiced in the field [61,62]. Our findings indicate that, at appropriate doses, the presence of metolachlor does not amplify the effects of green manure cultivation practices, and at the highest concentration, even though they alter some microbial responses, the interactions and microbiological quality are maintained.

The bacterial sensitivity can be partly explained by specific functional characteristics, a recurring situation when bacterial cells are active in natural communities [63]. The removal of metolachlor by remediation promotes bacterial alteration in the soil [64], a situation identified in areas with *A. sativa*, and partially explains the observed values. On the other hand, the decrease in indices in areas with this plant species without herbicide indicates a likely restriction of this microbial group associated with the plant. The defense of *A. sativa* against certain bacterial groups was confirmed by other studies with gene expression patterns [65].

Regarding *M. sativa*, it is likely that the positive interaction between the plant and the microbial community outweighs any potential effect of herbicide presence. Studies indicate that in the rhizosphere, between rhizobacteria and *M. sativa* plants, there is an active interaction at the level of extracellular amino acid exchange [66]. According to these authors, plant roots absorb the amino acids excreted by bacteria, and these bacteria absorb the amino acids excreted by *M. sativa* roots.

There is complexity in working with uncultivable and undetermined individuals due to the difficulty in measuring their functional potential and biotechnological applications. However, in our findings, there is a correlation between uncultivable and undetermined organisms in areas containing metolachlor (AM2 and MM2). The occurrence of possible bacterial genera involved in the biodegradation of the molecule was noticeable, as confirmed for *Streptomyces lydicus* [67], *Pseudomonas* sp. [68]., *Nitrososphaera*, *Nitrosospira*, *Sulfuricella*, *Gemmatimonas*, *Candidatus* sp., *Burkholderia*, *Bradyrhizobium*, *Accumulibacter*, *Entotheonella*, *Cupriavidus*, *Azoarcus*, *Thiobacillus*, and *Sideroxydans* [69].

The richness and diversity ratio of fungi increased in the presence of metolachlor, associated with green manures. The effects of the interaction between green manures and microorganisms, combined with climatic effects, are more relevant predictors in the final composition of the fungal community [70].

Symbiotic fungal species that colonize the roots of green manures are common, with reports of herbicide degradation such as atrazine. The interaction between the roots of green manures and fungi such as *Aspergillus niger*, *Phanerochaete* sp., and *Pleurotus* sp. enabled the addition of sugars that aided these microorganisms in breaking down the atrazine molecule [71].

Regarding *A. sativa*, the decrease in the abundance of unidentified microorganisms is associated with the selection pressure imposed by this species, reducing occurrence, and in a synergistic effect with the herbicide prevailing, however, in some families such as *Mortierellaceae*. The increase in the abundance of *Mortierellaceae* has been reported as a result of metolachlor bioremediation [72]. It helps explain the potential of *A. sativa* as a phytoremediator of this herbicide.

Some fungal specimens undergo broader impacts on the composition and structure of the community, both biotically through the suppressiveness of control agents such as *Papiliotrema* sp., *Humicola* sp., *Trichoderma* sp., and *Epicoccum* and abiotically through the presence of the herbicide molecule [73]. The genera *Humicola* sp., *Trichoderma* sp., *Pleurophragmium* sp., and *Saitozyma* sp. were the most abundant in soils of *A. sativa* under the effect of the highest dose of metolachlor, indicating possible plant–microorganism interaction in the phytoremediation process. These genera are involved in enzyme production and nutrient cycling directly in phytopathogen control [74] and have been observed as agents in herbicide decomposition [75]. Although we associate higher abundance values with degradation potential, we do not dismiss the fact that species with low percentages, such as *Trichoderma*, are widely known for metolachlor degradation [62]. In our study, *Trichoderma* was highlighted in all treatments; however, metolachlor application significantly altered the abundance of this fungus, increasing it in *A. sativa* roots and decreasing it in *M. sativa* roots. Thus, this variation serves as a good example that the association between fungi and roots of remediating species should be further explored so that agricultural practices using bioinputs can be more accurately directed.

## 5. Conclusions

*Avena sativa* and *Medicago sativa* were effective in reducing the residual concentration of metolachlor in the soil.

Remediation efficiency was particularly notable in areas treated with higher doses of the herbicide.

The application of metolachlor negatively affected the soil’s microbial biodiversity, reducing the uniformity and distribution of species. But the presence of *Medicago sativa* promoted greater richness and distribution of microbial species, even under herbicide pressure.

The presence of metolachlor affected the distribution and abundance of these families, but the presence of phytoremediating plants mitigated the effects.

## Figures and Tables

**Table 1:** List of treatments for evaluating the impact of metolachlor * on the genomics of rhizosphere microorganisms of *Avena sativa* and *Medicago sativa* plants used for herbicide residue phytoremediation.

Treatments	Phytoremediation Species	Herbicide	Dose (g/ha)
AM1	*Avena sativa*	Metolachlor	530.70
AM2	*Avena sativa*	Metolachlor	1061.40
MM1	*Medicago sativa*	Metolachlor	530.70
MM2	*Medicago sativa*	Metolachlor	1061.40
A-0	*Avena sativa*	-	-
M-0	*Medicago sativa*	-	-
NoM1	-	Metolachlor	530.70
NoM2	-	Metolachlor	1061.40
No-0	-	-	-

*/Application of Sequence^®^, containing 353.8 g/L of metolachlor and 265.7 g/L of glyphosate. No-0: soil samples collected from a plot without herbicide application or cultivation of the species. AM1 and AM2—*A. sativa* cultivated in soil with 530.7 g/ha, and 1061.4 g/ha of metolachlor, MM1, and MM2—*M. sativa* cultivated in soil with 530.7, and 1061.4 g/ha of metolachlor; A-0—*A. sativa* in soil without herbicide (control); M-0—*M. sativa* in soil without herbicide (control), NoM1, and NoM2—soil without cultivation and with 530.7 g/ha, and 1061.4 g/ha of metolachlor, and No-0—native Cerrado soil.

**Table 2:** Global ecological index of microbial diversity in *Avena sativa* and *Medicago sativa* areas, cultivated in soil with metolachlor herbicide residues.

*Avena sativa*						*Medicago sativa*			
	Unif ^1^	Richness	Dist. ^2^	Dom ^3^		Unif ^1^	Richness	Dist. ^2^	Dom ^3^
A0	0.71	104.0	3.3	0.92	M0	0.90	120.0	4.2	1.0
AM1	0.79	121.0	3.8	0.96	MM1	0.90	122.0	4.2	1.0
AM2	0.86	116.0	4.1	0.98	MM2	0.90	117.0	4.2	1.0
No-0	0.85	109.0	4.0	0.97	No-0	0.90	109.0	4.0	1.0

A0—*Avena sativa* in soil without herbicide (control), AM1 and AM2—*A. sativa* cultivated in soil with 530.7 and 1061.4 g/ha of metolachlor. No-0—native Cerrado soil. M0—*Medicago sativa* in soil without herbicide (control), MM1 and MM2—*M. sativa* cultivated in soil with 530.7 and 1061.4 g/ha of metolachlor. ^1^ Evenness: Pielou’s Evenness Index; Richness: Chao1 Index; ^2^ Species Distribution: Shannon’s Index. ^3^ Dominance/Diversity: Gini-Simpson Index.

**Table 3:** Ecological index of bacterial population in areas cultivated with *Avena Sativa* and *Medicago sativa*, subjected or not subjected to metolachlor application, as well as a native Cerrado area.

*Avena sativa*						*Medicago sativa*			
	Unif ^1^	Richness	Dist. ^2^	Dom ^3^		Unif ^1^	Richness	Dist. ^2^	Dom ^3^
A0	0.71	28.0	2.38	0.77	M0	0.94	44.0	3.57	0.96
AM1	0.90	44.0	2.40	0.94	MM1	0.94	41.0	3.51	0.95
AM2	0.93	40.0	3.43	0.95	MM2	0.90	39.0	3.32	0.94
No-0	0.95	42.0	3.30	0.96	No-0	0.94	42.0	3.53	0.96

A0—*A. sativa* in soil without herbicide (control), AM1 and AM2—*A. sativa* cultivated in soil with 530.70 and 1061.40 g/ha of metolachlor. No-0—native Cerrado soil. M0—*M. sativa* in soil without herbicide (control), MM1 and MM2—*M. sativa* cultivated in soil with 530.70 and 1061.40 g/ha of metolachlor. ^1^ Evenness: Pielou’s Evenness Index; Richness: Chao1 Index; ^2^ Species Distribution: Shannon’s Index. ^3^ Dominance/Diversity: Gini-Simpson Index.

**Table 4:** Abundance (%) of predominant families and genera of the bacterial community associated with *Avena sativa* and *Medicago sativa* with or without metolachlor residues and in the native Cerrado soil area.

	A-0	AM1	AM2	M-0	MM1	MM2	No-0
	------------------------------------- Family -------------------------------------						
Uncultivated	8.69	9.59	8.27	7.91	9.49	14.66	17.97
*Beijerinckiaceae*	4.42	4.09	4.16	1.84	3.4	3.25	1.65
*Bryobacteraceae*	1.84	1.75	1.28	*	*	*	3.60
*Burkholderiaceae*	10.63	7.80	5.12	4.75	5.20	6.46	1.89
Undetermined	6.51	7.91	9.04	4.90	6.97	7.27	9.63
*Ktedonobacteraceae*	*	*	*	0.54	0.81	0.72	5.68
*Micrococcaceae*	11.01	10.20	14.27	36.60	17.50	3.58	2.78
*Mycobacteriaceae*	3.82	2.58	3.26	1.91	2.82	1.83	2.76
*Solirubrobacteraceae*	4.89	4.33	5.47	3.20	4.57	4.74	11.91
*Sphingomonadaceae*	3.60	4.20	3.92	2.64	3.25	1.58	1.05
*Xanthobacteraceae*	8.17	7.90	12.56	7.68	7.32	17.74	12.85
	------------------------------------- Genus -------------------------------------						
Undetermined	47.12	40.62	32.40	31.00	32.89	38.73	34.45
Uncultivated	11.92	16.53	19.93	16.03	17.53	14.68	31.23
*Arthrobacter* sp.	0.71	1.19	0.53	*	*	*	1.78
*Baekduia* sp.	0.89	0.82	0.62	1.09	0.89	1.35	1.91
*Bradyrhizobium* sp.	2.48	2.70	4.78	3.46	2.78	3.55	2.75
*Massilia* sp.	2.13	1.56	2.06	3.49	2.22	1.95	0.30
*Methylobacterium* sp.	*	*	*	2.69	2.04	2.54	0.40
*Mycobacterium* sp.	1.53	2.29	1.59	3.06	2.3	2.89	2.58
*Pseudarthrobacter* sp.	3.47	0.80	0.46	1.78	1.65	2.46	0.06
*Sphingomicrobium* sp	2.01	2.69	1.31	2.67	2.96	2.89	0.82
*Streptomyces* sp.	2.54	1.25	0.89	1.68	1.01	2.16	1.58

A-0—*A. sativa* in soil without herbicide (control), AM1 and AM2—*A. sativa* cultivated in soil with 530.7 and 1061.4 g/ha of metolachlor. No-0—native Cerrado soil. M-0—*M. sativa* in soil without herbicide (control), MM1 and MM2—*M. sativa* cultivated in soil with 530.70 and 1061.40 g/ha of metolachlor. The symbol (*) represents the microbial group below the top 10 occurrences of microorganisms at the family and genus levels.

**Table 5:** Ecological index of the fungal population in areas cultivated with *Avena sativa* and *Medicago sativa*, whether or not they were subjected to metolachlor application, in addition to a native Cerrado area.

*Avena sativa*						*Medicago sativa*			
	Unif ^1^	Richness	Dist. ^2^	Dom ^3^		Unif ^1^	Richness	Dist. ^2^	Dom ^3^
A0	0.64	76.0	2.78	0.87	M0	0.81	76.0	3.52	0.95
AM1	0.67	77.0	2.91	0.90	MM1	0.79	81.0	3.48	0.94
AM2	0.79	76.0	3.40	0.95	MM2	0.82	78.0	3.58	0.96
No-0	0.75	67.0	3.17	0.93	No-0	0.75	67.0	3.17	0.93

A0—*A. sativa* in soil without herbicide (control), AM1 and AM2—*A. sativa* cultivated in soil with 530.70 and 1061.40 g/ha of metolachlor. No-0—native Cerrado soil. M0—*M. sativa* in soil without herbicide (control), MM1 and MM2—*M. sativa* cultivated in soil with 530.70 and 1061.40 g/ha of metolachlor. ^1^ Evenness: Pielou’s Evenness Index; Richness: Chao1 Index; ^2^ Species Distribution: Shannon’s Index. ^3^ Dominance/Diversity: Gini-Simpson Index.

**Table 6:** Abundance (%) of predominant families and genera of the fungi community associated with *Avena sativa* and *Medicago sativa* with or without metolachlor residues and in the native Cerrado soil area.

	A-0	AM1	AM2	M-0	MM1	MM2	No-0
	------------------------------------- Family -------------------------------------						
Undetermined	7.42	17.35	18.13	3.18	2.11	2.76	11.06
*Aspergillaceae*	10.46	7.41	12.18	10.49	6.01	9.55	21.14
*Chaetomiaceae*	6.65	4.77	3.74	8.58	8.55	13.69	6.91
*Cladosporiaceae*	3.65	4.43	5.01	13.06	14.61	2.07	3.71
*Cunninghamellaceae*	1.24	0.49	2.77	1.08	0.51	2.38	7.88
*Didymellaceae*	12.38	4.57	13.19	13.84	6.06	7.06	3.25
*Filobasidiaceae*	*	*	*	2.73	7.01	2.93	0.01
*Hypocreaceae*	9.22	1.37	1.03	0.46	2.05	7.37	8.44
*Mortierellaceae*	3.32	19.57	4.87	*	*	*	0.00
*Nectriaceae*	12.13	4.21	6.57	28.88	11.79	12.45	18.6
*Phaeosphaeriaceae*	1.42	1.38	2.96	*	*	*	2.29
*Rhynchogastremataceae*	*	*	*	1.49	20.00	4.73	0.26
	---------------------------------------- Genus ----------------------------------------						
*Cladosporium* sp.	13.06	14.61	2.07	19.52	24.94	31.39	3.71
*Epicoccum* sp.	3.64	4.56	3.50	5.23	1.53	8.57	1.69
*Fusarium* sp.	28.76	11.57	10.89	11.94	3.44	6.18	18.19
*Humicola* sp.	5.41	6.30	10.43	4.90	2.28	1.99	1.42
*Undetermined*	14.72	6.42	12.67	*	*	*	28.41
*Mortierella* sp.	*	*	*	3.32	19.57	4.87	0.00
*Papiliotrema* sp.	1.49	20.0	4.73	*	*	*	0.26
*Penicillium* sp.	10.06	5.82	9.08	9.32	6.40	10.89	11.08
*Pleurophragmium* sp.	1.29	2.04	7.19	1.63	3.44	2.21	0.01
*Saitozyma* sp.	1.10	2.12	6.41	*	*	*	0.09
*Trichoderma* sp.	0.46	1.93	7.37	9.20	1.37	1.03	8.44

A-0—*A. sativa* in soil without herbicide (control), AM1 and AM2—*A. sativa* cultivated in soil with 530.70 and 1061.40 g/ha of metolachlor. No-0—native Cerrado soil. M-0—*M. sativa* in soil without herbicide (control), MM1 and MM2—*M. sativa* cultivated in soil with 530.70 and 1061.40 g/ha of metolachlor. The symbol (*) represents the microbial group below the top 10 occurrences of microorganisms at the family and genus levels.

## Data Availability

The original contributions presented in the study are included in the article, further inquiries can be directed to the corresponding author/s.

## References

[1] Nath C.P., Singh R.G., Choudhary V.K., Datta D., Nandan R., Singh S.S. (2024). Challenges and Alternatives of Herbicide-Based Weed Management. Agronomy.

[2] Lima A.d.C., Mendes K.F. (2020). Variable Rate Application of Herbicides for Weed Management in Pre- and Postemergence.

[3] Bhardwaj L., Reddy B., Nath A.J., Dubey S.K. (2024). Influence of herbicide on rhizospheric microbial communities and soil properties in irrigated tropical rice field. Ecol. Indic..

[4] Singh A.K., Pandey A.K. (2024). Chapter 9—Exploitation of mycometabolites in weed management: Global scenario and future application. Entrepreneurship with Microorganisms.

[5] Li X., Zhang H., Sun M., Xu N., Zhao M. (2020). Land use change from upland to paddy field in Mollisols drives soil aggregation and associated microbial communities. Appl. Soil Ecol..

[6] Thiour-Mauprivez C., Martin-Laurent F., Calvayrac C. (2019). Effects of herbicide on non-target microorganisms: Towards a new class of biomarkers?. Sci. Total Environ..

[7] Hachisu S. (2021). Strategies for discovering resistance-breaking, safe, and sustainable commercial herbicides with novel modes of action and chemotypes. Soc. Chem. Ind..

[8] Adomako M. (2016). Effect of Some Commonly Used Herbicides on Soil Microbial Population. J. Environ. Earth Sci..

[9] Hussain Lone A., Raverkar K.P., Pareek N. (2014). In-vitro effects of herbicides on soil microbial communities. Bioscan.

[10] Wilfred K., Kwame Dzomeku I., Xorse K.J. (2020). Efficacy of pre-emergence and post-emergence herbicides for weed management in groundnut (*Arachis hypogaea* L.) production in Guinea Savannah. Int. J. Sci. Res. Manag. (IJSRM).

[11] Yilmaz G., Feruzan D.A.N. (2013). Phytotoxic effects of herbicide Attribut and surfactant BioPower on the root, stem, and leaf anatomy of Triticum aestivum ‘Pehlivan’. Turk. J. Bot..

[12] Atwood D., Paisley J.C. (2017). Pesticides Industry Sales and Usage: Market Estimates.

[13] Kouame K.B.J., Bertucci M.B., Savin M.C., Bararpour T., Steckel L.E., Butts T.R., Willett C.D., Machado F.G., Roma-Burgos N. (2022). Resistance of Palmer amaranth (*Amaranthus palmeri*) to Metolachlor in the midsouthern United States. Weed Sci..

[14] Silva Q.M., Palmieri M.J., Andrade-Vieira L.F. Effects of a S-Metolachlor Based Herbicide on Two Plant Models: *Zea mays* L. and *Lactuca sativa* L.. Preprint.

[15] Da Silva M.S., Furtado J.A.L., Castro J.Q., dos Santos L.L., Almeida E.I.B., de Oliveira L.B.T., Sousa W.d.S., Araújo R.C.d.A. (2022). Weed control and selectivity of different pre-emergence active ingredients in a soybean crop. Agron. Colomb..

[16] MAPA, Ministério da Agricultura e Pecuária (2023). Plano Setorial de Mitigação e de Adaptação às Mudanças Climáticas para a Consolidação de uma Economia de Baixa Emissão de Carbono na Agricultura.

[17] Grădilă M., Jalobă D., Ciontu V., Erban M., Petcu V. (2021). Research regarding weed control in grain legumes crops. Sci. Pap. Ser. A Agron..

[18] Chauhan B.S., Gill G.S., Preston C. (2007). Timing and Dose of Metolachlor Affect Rigid Ryegrass (*Lolium rigidum*) Control in Wheat. Weed Technol..

[19] Mesnage R., Panzacchi S., Bourne E., Mein C.A., Perry M.J., Hu J., Chen J., Mandrioli D., Belpoggi F., Antoniou M.N. (2022). Glyphosate and its formulations Roundup Bioflow and RangerPro alter bacterial and fungal community composition in the rat caecum microbiome. Front. Microbiol..

[20] Curran W.S. (2016). Persistence of herbicides in soil. Crops Soils Mag..

[21] Sanyal D., Yaduraju N.T., Kulshestha G. (2008). Metolachlor persistence in laboratory and field soils under Indian tropical conditions. J. Environ. Sci. Health Part B Pestic. Food Contam. Agric. Wastes.

[22] Dinelli G., Accinelli C., Vicari A., Catizone P. (2000). Comparison of the Persistence of Atrazine and Metolachlor under Field and Laboratory Conditions. J. Agric. Food Chem..

[23] Aguiar L.M., de Freitas Souza M., de Laia M.L., de Oliveira Melo J., da Costa M.R., Gonçalves J.F., Dos Santos J.B. (2020). Metagenomic analysis reveals mechanisms of atrazine biodegradation promoted by tree species. Environ. Pollut..

[24] Barroso G.M., Dos Santos E.A., Ribeiro Pires F., Galon L., Cabral G.M., Dos Santos J.B. (2023). Phytoremediation: A green and low-cost technology to remediate herbicides in the environment. Chemosphere.

[25] Oliveira M.C., Osipitan O.A., Begcy K., Werle R. (2020). Cover crops, hormones and herbicides: Priming an integrated weed management strategy. Plant Sci..

[26] Scavo A., Mauromicale G. (2020). Integrated Weed Management in Herbaceous Field Crops. Agronomy.

[27] Silva T.S., Arneson N.J., DeWerff R.P., Smith D.H., Silva D.V., Werle R. (2023). Preemergence herbicide premixes reduce the risk of soil residual weed control failure in corn. Weed Technol..

[28] Vasileiou M., Vasileiou M., Kyrgiakos S., Kleisiari L., Kleftodimos C., Vlontzos G., Belhouchette G., Pardalos P.M. (2024). Transforming weed management in sustainable agriculture with artificial intelligence: A systematic literature review towards weed identification and deep learning. Crop Prot..

[29] Quince C., Walker A.W., Simpson J.T., Loman N.J., Segata N. (2017). Shotgun metagenomics, from sampling to analysis. Nat. Biotechnol..

[30] Barroso G.M., dos Santos J.B., de Oliveira I.T., Nunes T.K.M.R., Ferreira E.A., Pereira I.M., Silva D.V., Souza M.F. (2020). Tolerance of Bradyrhizobium sp. BR 3901 to herbicides and their ability to use these pesticides as a nutritional source. Ecol. Indic..

[31] Mauser K.M., Brühl C.A., Zaller J.G. (2024). Herbicide Effects on Nontarget Organisms, Biodiversity and Ecosystem Functions. Encycl. Biodivers..

[32] Scheepmaker J.W.A., van de Kassteele J. (2011). Effects of chemical control agents and microbial biocontrol agents on numbers of non-target microbial soil organisms: A meta-analysis. Biocontrol Sci. Technol..

[33] Liao H., Li X., Yang Q., Bai Y., Wen C., Liu C., Chen Z., Tang J., Yu Z., Che J. (2021). Herbicide selection promotes antibiotic resistance in soil microbiomes. Mol. Biol. Evol..

[34] Rose M.T., Cavagnaro T.R., Scanlan C.A., Rose T.J., Vancov T., Kimber S., Kennedy I.R., Kookana R.S., Zwieten L.V. (2016). Impact of herbicides on soil biology and function. Adv. Agron..

[35] Gonçalves dos Santos H., Jacomine P.K.T., Cunha dos Anjos L.H., de Oliveira V.A., de Oliveira J.B., Coelho M.R., Lumbreras J.F., Ferreira Cunha T.J. (2006). Sistema Brasileiro de Classificação de Solos.

[36] Primavesi A.C., De Andrade Rodrigues A., Godoy R. (2000). Recomendações Técnicas para o Cultivo de Aveia.

[37] Rassini J.B. (1999). Alfafa (Medicago sativa L.): Estabelecimento e Cultivo no Estado de São Paulo.

[38] Ghosh P.K., Bandyopadhyay K.K., Wanjari R.H., Manna M.C., Misra A.K., Mohanty M., Subba Rao A. (2014). Legume Effect for Enhancing Productivity and Nutrient Use-Efficiency in Major Cropping Systems—An Indian Perspective: A Review. J. Sustain. Agric..

[39] Prasad R. (2009). Efficient fertilizer use: The key to food security and better environment. J. Trop. Agric..

[40] Geronime E., Aparicio V. (2022). Changes in soil pH and addition of inorganic phosphate affect glyphosate adsorption in agricultural soil. Eur. J. Soil Sci..

[41] Guiasu R.C., Guiasu S. (2010). The Rich-Gini-Simpson quadratic index of biodiversity. Nat. Sci..

[42] Mouillot D., Lepretre A. (1999). A comparison of species diversity estimators. Rest Popul. Ecol..

[43] Wang R., Zhang R., Song P., Liu S., Li Y., Li H. (2023). Diversity and Distribution of 18 Cephalopod Species, and Their Link with Some Environmental Factors in the NW Pacific. Diversity.

[44] Brondi S.H., Macedo A.N., Vicente G.H., Nogueira A.R. (2011). Evaluation of the QuEChERS Method and Gas Chromatography–Mass Spectrometry for the Analysis of Pesticide Residues in Water and Sediment. Bull. Environ. Contam. Toxicol..

[45] Kemmerich M. (2017). Resíduos de Agrotóxicos em Ameixa, Maçã, pera e Pêssego: Desenvolvimento de Métodos de Análise e Monitoramento. Ph.D. Thesis.

[46] Yu L., Van Eerd L.L., O’Halloran I., Sikkema P.H., Robinson D.E. (2015). Response of four fall-seeded cover crops to residues of selected herbicides. Crop Prot..

[47] Jiang S., Wang J., Xie P., Tan L., Han L. (2023). Discontinuous low temperature stress and plant growth regulators during the germination period promote roots growth in alfalfa (*Medicago sativa* L.). Plant Physiol. Biochem..

[48] Paul S.K., Mazumder S., Naidu R. (2024). Herbicidal weed management practices: History and prospects of nanotechnology in an eco-friendly crop production system. Heliyon.

[49] Mackay J.E., Bernhardt L.T., Smith R.G., Ernakovich J.G. (2023). Seedage and pesticide seed treatments have distinct effects on soil microbial diversity and function. Soil Biol. Biochem..

[50] Pathak H.K., Seth C.S., Chauhan P.K., Dubey G., Singh G., Jain D., Upadhyay S.K., Dwivedi P., Khoo K.S. (2024). Recent advancement of nano-biochar for the remediation of heavy metals and emerging contaminants: Mechanism, adsorption kinetic model, plant growth and development. Environ. Res..

[51] Wołejko E., Kaczyński P., Łozowicka B., Wydro U., Borusiewicz A., Hrynko I., Konecki R., Snarska K., Dec D., Malinowski P. (2017). Dissipation of Metolachlor in plant and soil and effect on enzymatic activities. Environ. Monit. Assess..

[52] Borowik A., Wyszkowska J., Kucharski J., Bacmaga M., Tomkiel M. (2017). Response of microorganisms and enzymes to soil contamination with a mixture of terbuthylazine, mesotrione, and Metolachlor. Environ. Sci. Pollut. Res..

[53] Munoz A., Koskinen W.C., Cox L., Sadowsky M.J. (2011). Biodegradation and Mineralization of Metolachlor and Alachlor by Candida xestobii. J. Agric. Food Chem..

[54] Morales-Becerra C.E., Ortiz-Rojas L.Y., Chaves-Bedoya G. (2023). Assessment of Burkholderia glumae control in rice (*Oryza sativa*) FEDEARROZ 67, using silver nanoparticles (AgNPs) under greenhouse conditions. Revista Colombiana de Ciencias Hortícolas.

[55] Qin P., Hu L., Liu Y., Hu X., Zhang X., Rosado A.S., Wei G., Chen C. (2024). Responses of soil microbial communities and nutrient dynamics under continuous alfalfa (*Medicago sativa* L.) cultivation. Soil Ecol..

[56] Lyra W.d.S., da Silva E.C., de Araújo M.C.U., Fragoso W.D., Veras G. (2010). Classificação periódica: Um exemplo didático para ensinar análise de componentes principais. Química Nova.

[57] El Jaouhari M., Damour G., Tixier P., Coulis M. (2023). Glyphosate reduces the biodiversity of soil macrofauna and benefits exotic over native species in a tropical agroecosystem. Basic Appl. Ecol..

[58] Jousset A., Bienhold C., Chatzinotas A., Gallien L., Gobet A., Kurm V., Hol W.H.G. (2017). Where less may be more: How the rare biosphere pulls ecosystems strings. ISME J..

[59] Vryzas Z., Papadakis E.N., Oriakli K., Moysiadis T.P., Papadopoulou-Mourkidou E. (2012). Biotransformation of atrazine and metolachlor within soil profile and changes in microbial communities. Chemosphere.

[60] Yang R., Song S., Chen S., Du Z., Kong J. (2022). Adaptive evaluation of green manure rotation for a low fertility farmland system: Impacts on crop yield, soil nutrients, and soil microbial community. Catena.

[61] Wang J., Zhang L., Fan J., Wen Y. (2017). Impacts of Rac- and S-metolachlor on cyanobacterial cell integrity and release of microcystins at different nitrogen levels. Chemosphere.

[62] Bernat P., Jasinska A., Niedziałkowska K., Słaba M., Ro’zalska S., Paraszkiewicz K., Sas-Paszt L., Heipieper H.J. (2023). Adaptation of the metolachlor-degrading fungus Trichoderma harzianum to the simultaneous presence of low-density polyethylene (LDPE) microplastics. Ecotoxicol. Environ. Saf..

[63] Bickel S., Or D. (2021). The chosen few variations in common and rare soil bacteria across biomes. ISME J..

[64] Elsayed O.F., Maillard E., Vuilleumier S., Imfeld G. (2014). Bacterial communities in batch and continuous-flow wetlands treating the herbicide S-metolachlor. Sci. Total Environ..

[65] Emamifar S., Abolmaali S., Sohrabi S.M., Mohammadi M., Shahmohammadi M. (2020). Molecular characterization and evaluation of the antibacterial activity of a plant defensin peptide derived from a gene of oat (*Avena sativa* L.). Phytochemistry.

[66] Smirnova I., Sadanov A., Baimakhanova G., Faizulina E., Tatarkina L. (2022). Metabolic interaction at the level of extracellular amino acids between plant growth-promoting rhizobacteria and plants of alfalfa (*Medicago sativa* L.). Rhizosphere.

[67] Wang Z., Wang Z., Solanki M.K., Yu Z.-X., Anas M., Dong D.-F., Xing Y.-X., Malviya M.K., Pang F., Li Y.-R. (2021). Genome Characteristics Reveal the Biocontrol Potential of Actinobacteria Isolated From Sugarcane Rhizosphere. Front. Microbiol..

[68] Viegas C.A., Costa C., André S., Viana P., Ribeiro R., Moreira-Santos M. (2012). Does S-Metolachlor affect the performance of Pseudomonas sp. strain ADP as a bioaugmentation bacterium for atrazine-contaminated soils?. PLoS ONE.

[69] Feng G., Xie T., Wang X., Bai J., Tang L., Zhao H., Wei W., Wang M., Zhao Y. (2018). Metagenomic analysis of microbial community and function involved in cd-contaminated soil. BMC Microbiol..

[70] Tedersoo L., Bahram M., Polme S., Koljalg U., Yorou N.S., Wijesundera R., Suija A. (2014). Global diversity and geography of soil fungi. Science.

[71] Henn C., Monteiro D.A., Boscolo M., da Silva R., Gomes E. (2020). Biodegradation of atrazine and ligninolytic enzyme production by basidiomycete strains. BMC Microbiol..

[72] Li X., Li Y., Zhao X., Zhang X., Zhao Q., Wang X., Li Y. (2019). Restructured fungal community diversity and biological interactions promote metolachlor biodegradation in soil microbial fuel cells. Chemosphere.

[73] Li X., Omolehin O., Hemmings G., Tseng H.T., Taylor A., Taylor C., Kong P., Daughtrey M., Luster D., Gouker F. (2023). Boxwood phyllosphere fungal and bacterial communities and their differential responses to film-forming anti-desiccants. BMC Microbiol..

[74] Ghareeb Y., Belal R., El-Khateeb E.B., El-Khateeb NM M. (2024). Utilização da biossíntese de nanomateriais como agentes biológicos para o controle de doenças transmitidas pelo solo em plantas de pimenta: Nematóides das galhas e fungos da podridão radicular. BMC Plant Biol..

[75] Bhatt P., Verma A., Gangola S., Bhandari G., Chen S. (2021). Microbial glycoconjugates in organic pollutant bioremediation: Recent advances and applications. Microb. Cell Fact..

